# Acknowledgment to Reviewers of *Healthcare* in 2020

**DOI:** 10.3390/healthcare9010103

**Published:** 2021-01-19

**Authors:** Healthcare Editorial Office

**Affiliations:** MDPI AG, St. Alban-Anlage 66, 4052 Basel, Switzerland

Peer review is the driving force of journal development, and reviewers are gatekeepers who ensure that *Healthcare* maintains its standards for the high quality of its published papers. Thanks to the cooperation of our reviewers, in 2020, the median time to first decision was 18 days and the median time to publication was 38 days. The editors would like to express their sincere gratitude to the following reviewers for their precious time and dedication, regardless of whether the papers were finally published:
Abdul Wahab, OmarLin, Chih-MingAbe, TakumiLin, Feng-ChengAbel, LarryLin, Gen-MinAbuin, VanesaLin, HualiangAcharya, ArpanLin, Jin-DingAddison, CliftonLin, Yen-KoAdekoya, Timothy O.Linhardt, TinaAdeyinka, Adewuyi AyodeleLittle, JulianAgarwal, PoonamLiu, FilipeAggarwal, MeghaLiu, JianyeAgudelo, AndrésLiu, LukaiAhluwalia, MeenakshiLiu, TaoAhmad, ShakilLiu, XiaoqiangAhmed, FaheemuddinLizcano Casas, DavidAhmed, MohiuddinLizot, MauroAla-Ruona, EsaLloret, DanielAlcantud, José CarlosLo Sardo, FedericaAldieri, LuigiLo, Wei-ShuoAl-Ezzi, MinanLogger, JadeAliaño González, María JoséLombardo, GiorgioAlkhachroum, AyhamLombardo, MauroAlkhateeb, AbedalrhmanLong, BradleyAllegaert, KarelLoos, EugèneAllen, HerbertLópez Entrambasaguas, Olga MariaAllu, Prasanna K.R.López, DanielAlmeida, SaraLópez-García, SergioAlmendra-Pegueros, RafaelLopez-Liria, RemediosAlmond, HelenLópez-López, DanielAlmoshmosh, NadimLópez-Pintor Muñoz, Rosa MaríaAmiri, SolmazLozano-Lorca, MacarenaAmmirabile, AngelaLucchese, GianlucaAmnie, AsratLuis Ubago, JoseAmpollini, LucaLukaschek, KarolineAn, MinjeongLumme, SonjaAnanthakrishnan, Soundaram JeevarathinamLuo, ZhenAncel, JulienLupattelli, AngelaAndersson, HenrikLygo-Baker, SimonAndrade, Vanda MariaLymberopoulos, Dimitrios K.Andrew, LesleyLysecki, DaveAndriessen, KarlMa, EnboAngel, BárbaraMa, HongAnna Poulia, KalliopiMa, LiangAnsmann, LenaMa, ShuanggeAparicio-Martinez, PilarMa, WeiAparisi, DavidMäättänen, IlmariApte, NikhilMabire, Jean-BernardAquavella, James V.Macaluso, FilippoArakaki, TatsuyaMaccioni, Alessandro FioriArguelles-Cruz, Amadeo J.Macedo, João CarlosAridi, Yasmine S.Machado, PedroAriza, Maria EugeniaMacías Aranda, FernandoArmstrong, MichelleMacias-Diaz, Jorge E.Arness, DavidMacijauskienė, JūratėArnold, Amy C.Mackenstedt, UteArooj, FatimaMacPhee, MauraArora, GunjanMagalhães, BrunoArroyo Pardo, EduardoMagori, KrisztianAshok, AshwinMaisha, BuumaAsif, MuhammadMakaryus, RanyAssandri, FaustoMalave-Llamas, KarloAuerbach, SanfordMalvè, MauroAugustynek, MartinManandhar, RejinaAustveg, BeritMandić-Rajčević, StefanAwan, HaroonMangla, AnkitAwedat, KhalfallaMankowska-Wierzbicka, DorotaAyanou, TilahunManning, JimmieAzzolino, DomenicoManuel, Rodríguez-HuguetBaccouche, AsmaManzo, CiroBacevičienė, MiglėMapelli, MassimoBadanta Romero, BárbaraMarasso, Simone LuigiBaer, ThomasMarcu, SilviaBaeyens, Jean-PierreMarie, Jean-PaulBaghdadi, Ziad D.Marotta, ClaudiaBahari-Chopiuk, NasrinMarques-Sanchez, PilarBailey, CatherineMárquez, María Del Mar SimónBakaniene, IndreMartell, KevinBallini, AndreaMartin, EricBalsevičius, TomasMartin, Richard H.Balzarro, MatteoMartín-Conty, José LuisBarcikowska, ZofiaMartínez Ramón, Juan PedroBarone, MicheleMartinez Ruiz, Maria AngelesBarros, Joana M.Martinez, UrsulaBartniczak, BartoszMartínez-Galiano, Juan MiguelBartosiewicz, AnnaMartínez-González-Moro, IgnacioBassett, DarrylMartinez-Isasi, SantiagoBassi, GiuliaMartínez-Isasi, SantiagoBasu, Jayasree (Joy)Martínez-Martinez, JesúsBasu, ParthaMartínez-Rodríguez, AliciaBatchelor, PaulMartini, MicheleBathula, Chandra SekharMartini, RoseBattineni, GopiMartins, Felipe José AidarBaumann, KlausMARTOS-GARCIA, RAULBaumgarden, JosephMarverti, GaetanoBaylina, PilarMaselli, FilippoBegoray, DeborahMason, LauraBelcher, John R.Mather, CareyBell, SimonMatikonda, SiddharthBelousova, VictoriaMatłosz, PiotrBelteki, GusztavMatranga, DomenicaBelzunegui-Eraso, AngelMatsuda, AkiraBem, AgnieszkaMatsusaki, TakashiBender, Hans-UlrichMatta, JoaneBendick, MarcMattsson, JanetBennardo, FrancescoMatud, PilarBennett, AnnemarieMauceri, ManuelaBennett, ChristopherMaurya, ShailendraBenore, EthanMawani, AminBento, FabioMayner, LidiaBerezin, AlexanderMayor, David L.Bergefurt, LisanneMazur, JoannaBerry, William R.Mazzucato, MonicaBerteau, Jean-PhilippeMcColley, SusannaBEZU, LucilliaMcDonald, DavidBhuiyan, AzadMcDowell, LizBian, JunsongMcGorm, KellyBidmon, SonjaMcGrath, RyanBindels, JacquesMcgregor, NeilBingham, Jennifer M.McKinley, ErinBith-Melander, PollieMdegela, MselengeBlakely, Andrew M.Meagher, KelseyBoaz, AnnetteMehta, Ambereen KurwaBöckerman, Petri RikhardMeisinger, ChristaBogacki, JanMejzlik, JanBohannon, Richard W.Mekhemar, MohamedBolinger, ChristopherMendes, DiogoBonavina, LuigiMendoza-Nuñez, Victor ManuelBono, FilippaMenvielle, LoickBooth, AnneMeyer, Thomas J.Borghi, FrancescaMicha, DimitraBose, TanimaMichaels, NatalieBóta, AndrásMichalski, GrzegorzBotelho, Raquel Braz AssuncaoMiglo, AntonBotting, NicolaMihailidou, Anastasia SusieBotts, RyanMilanowski, LukaszBourne, AllisonMiletta, MariaBoussios, StergiosMira, José JoaquínBowen, DeborahMiranda Duro, María Del CarmenBowling, Heather L.Mita, KojiBracci, MassimoMiyama, TakeshiBregenzer, AnitaMoacir Godoy, MoacirBright, Candace ForbesMohammadi, EhsanBroderick, Tom L.Mohsen, AttayebBrown, Cary A.Moir, FionaBrown, Ronald B.Mokhlespour Esfahani, Mohammad ImanBruzzese, DarioMoldan, BedřichBucci, MarcoMolero, PatricioBuchta, ChristophMolin, FredrikBueno-Silva, BrunoMolina-Mula, JesúsBukhsh, Faiza AllahMolina-Sánchez, HoracioBurch, SarahMøller, GrithBurger, Divan AristoMonaco, Maria Grazia LourdesBurrascano, JosephMonahan, KathleenBusu, CristianMonfort-Pañego, ManuelBuzzi, MarinaMonsuez, Jean-JacquesByeon, HaewonMontanes, AntonioByrne, MargretMonteagudo Chiner, PabloCabrera-Chávez, FranciscoMonteiro, CristinaCaffò, AlessandroMonteiro, Samuel José FonsecaCafolla, DanieleMoon, SangheeCai, YingyunMorales Suárez-Varela, María M.Cairncross, CarolynMorales-Polo, CarlosCalcagni, GiulioMoral-García, José EnriqueCalvo-Lobo, CésarMoran, JoseCambray, SerafíMoreira, Mário W. L.Campagna, SaraMoreira, Mário Wedney De LimaCampmans-Kuijpers, Marjo JeMorenilla Jimeno-Morenilla, AntonioCañadas-De La Fuente, Guillermo A.Moreno, DaniCandemir, SemaMorikawa, TakamichiCAO, WangnanMoss, MarkCao, YangMotamed, CyrusCapitman, John AmsonMou, XichenCarboni, LuciaMourão, Carlos FernandoCarle, FlaviaMoussas, Vassilios C.Carlin, EmmaMüller-Tasch, ThomasCaron, RosemaryMulyasari, FarahCassetta, MicheleMunday, KarenCastellanos, Miguel ÁngelMurff, Sharon H.Castelli, LuciaMustacchi, GiorgioCastuera, Ruth JiménezMutlu, RahimCatalano Iniesta, LeonardoMutoro, Antonina N.Catalano, AntoninoMyles, Ian A.Cauli, OmarMyśliwiec, HannaCernuda, José A.Myszkowska-Ryciak, JoannaCésar Cavalcanti Roza, VálberNadhim, Evan A.Chan, Alan H. S.Nagarajan, PrabakaranChang, Chien-ChungNagwekar, JanhaviChang, Ke-VinNajjar, MohammadChaparro Rico, Betsy D.M.Nakajima, TakahitoChase, P. BryantNakazawa, MinatoChatterjee, SampurnaNam, TaewooChatterjee, SatabdiNapoli, ChristianChen, ChaoyangNardella, ElizabethChen, Chun-JungNarkhede, MayurChen, GongNaruse, TakashiChen, MinjianNash, CarolChen, SuiyaoNaskar, ShovanChen, Tsung-PoNathans, LauraCheng, Juei-TangNavarro Flores, EmmanuelCheong, Kang HaoNavas Sanchez, Maria PatriciaCheruvallil-Contractor, SariyaNegri, AttàChien, Chun-RuNemec, JurajChilaka, Marcus A.Németh, LászlóChiu, Yong-HuNemeth, Lynne S.Chmielinski, PawelNestares Pleguezuelo, Maria TeresaCho, Hyun-JaeNestrasil, IgorCho, SunghyounNewhouse, IanChoi, Im-RakNewman, John F.Choi, MyeongjinNewson, LisaChoi, SungyongNg, Qin XiangChoi, Won SukNgo, HanhChoo, HyojungNguyen, Hoang LongChoudhuri, AvikNiakšu, OlegasChruściel, PawełNie, PengChrysanthakopoulos, Nikolaos A.Nievas Soriano, Bruno JoséChtourou, HamdiNifli, Artemissia-PhoebeChu, XiangpingNishiyama, OsamuChuang, Hung-YiNjoku, AnuliChung, Hee-ChunNoble, MichaelChung, Kyung-YongNoblin, AliceCiarambino, TizianaNodera, HiroyukiCicciù, MarcoNojima, HiroyukiCingolani, MarianoNolan, ClodaghClari, MarcoNolwenn, LE MEURClemente-Suárez, Vicente JavierNoren, HasmunCobos-Puc, LuisNoushi, NioushahCoelho, Pedro Miguel VieiraNovío, SilviaCohen, Harold C.Nucera, RiccardoColgan, NiallNunes, Ana IsabelCollart-Dutilleul, Pierre-YvesNunnery, DanielleComacchio, CarlaO’Neal, SuzanneConde, BebianaOba, ShinoConson, MassimilianoObrero Gaitán, EstebanCoombs, Christopher KOdo, NnaemekaCoombs, Lorinda AOehring, DanielaCope, MichaelOhta, RyuichiCori, LilianaOjong, NathanaelCorrea, MaríaOkuda, MasayukiCosta, Emilia MartinsOliveira, JorgeCosta, SebastianoOlshansky, Stuart J.Coste, JérômeOlszewska-Guizzo, AgnieszkaCoussens, ScottO’Malley, LucyCoutts, KimOnagbiye, SundayCrippa, MatteoOnishi, HirotakaCristófol-Rodríguez, Francisco-JavierOno, YukoCronkite, Ruth C.Oppi, ChiaraCrowe, KellyOrtmeyer, Heidi K.Cruz, EdmanuelOwari, YutakaCruz-Fuentes, Carlos S.Padilla, MarielaCurado, AntónioPal Choudhuri, ShreoshiCzekierdowski, ArturPalazón-Bru, AntonioCzosnyka, MarekPalmer, JoeDąbrowski, WojciechPan, PengminDalgleish, MatPan, YijunDamiani, GiovanniPan, YushanDantas, Mario A.R.Parnianpour, MohamadDanvers, Alexander F.Parra-González, María ElenaDaoust, Jean-FrancoisParrish, RichardDapena, Alberto DiazPassarelli, Pier CarmineDash, BirajaPatel, DevenDatema, T. A. M.Patel, Jai N.D’auria, EnzaPatil, AshokDavlyatov, GanisherPato, LúciaDe Berardis, DomenicoPatricia, Concheiro-MoscosoDe Graaf, PetraPavlik, Edward J.De Haro, María Reina GranadosPedullà, LudovicoDe La Fuente-Solana, Emilia I.Pegado, ElsaDe La Torre-Montero, Julio-CésarPeñalba, AliciaDe Molon, Rafael ScafPeña-Sánchez, Juan-NicolásDe Re, VallìPenlington, ChrisDe Rosis, SabinaPenmetsa, Gopi K.De Sousa, Bruno MacedoPennington, Colin G.De Wilde, KatrienPentaris, PanagiotisDeevska, GerganaPérez Corrales, JorgeDel Giorno, RosariaPérez- De La Cruz, SagrarioDel Rio, A. BelenPérez Marfil, Maria NievesDe-la-Cruz-Torres, BlancaPérez, Ana María PalominoDelgado-Losada, María LuisaPérez-Bermejo, MarcelinoDelitala, AlessandroPérez-Ros, PilarDelmonico, LucasPerri, GianluigiDeng, WuPester, AndreasDenis, FredericPethick, JamesDenkenberger, DavidPetroianu, AndyDhir, AmandeepPetzold, GuillermoDi Battista, SilviaPhan, TungDi Martino, FerdinandoPiccoli, Giorgina BarbaraDi Venere, DanielaPietrangelo, TizianaDiAngelo, Denis J.Pires, CarlaDias, Cláudia SilvaPirozzi, FeliceDibaba, DanielPisanu, ClaudiaDidier Germain, ArnaudPitter, Janos G.Diethe, TomPlans-Rubi, PedroDiglio, AntonioPolanska, KingaDing, MengPoldermans, DonDiogo, ElisetePomara, CristoforoDirkson, AnnePond, DimityDisney, HeatherPorrovecchio, AlessandroDobrowolska, BeataPosada-Quintero, Hugo F.Dolezel, Diane MPottie, KevinDolores, VillalobosPownall, SueDomagala, AlicjaPrezoto, Benedito C.Donadu, MatthewPrieto, LeonelDonevant, Sara BellePrincipe, Maria Ilaria DelDonovan, Robert J.Prior, SarahDonta, SamuelPriyadarshini, AnushreeDopelt, KerenProcter, PaulaDrawert, BrianPrunty, Leesa M.Dreher, Mark L.Prunuske, AmyDuguez, StephaniePryss, RüdigerDumitrache, LilianaPu, BoDziedzic, ArkadiuszPulle, Ranjeev ChrysanthEhrenpreis, Eli DPurdom, TroyEitaro, KodaniQi, GuanqiuEl Mobadder, MarwanQiu, JianbinEleftheriadis, TheodorosQuide, YannElgendy, MostafaQuinn, Megan A.Elkins, JeananneRadicic, DraganaEndo, MakotoRafales, Cristina BonastreEriksson, CharliRahal, Rima-MariaErnesto, Cortés-CastellRahi, BernaEstrada, VicenteRamachandran, SujithExbrayat, Jean-MarieRambaud, Salvador CruzFaita, FrancescoRamirez Lopez, Leonardo JuanFajarda, OlgaRamirez, BernardoFarokhi, Moshtagh R.Ramirez-Baena, LuciaFarronato, GiampietroRamiro-Cortijo, DavidFauzi, IlianaRamli, Nurul NadiaFeatherstone, JDRan, JinjunFelipe, José LuisRanjan, KishuFernandes, LiseteRasool, Sama FaizFernandez Salinero, SamuelRather, IrfanFernández, María Dolores RuizRauf, AbdulFernández-Alcántara, ManuelRauschnabel, PhilFernández-Martínez, ElenaRavine, Terrence J.Fernández-Martínez, EliaRawla, PrashanthFerrara, PietroRaya-González, JavierFigueiredo, DanielaRego, PaulaFink, AstridReid, ScottFirmino, HoracioReif, Kathryn E.Fischer, TatjanaReis, PedroFlasher, ReneeReiter, E. MirandaFlood, MichelleRennels, Jennifer L.Flood-Grady, ElizabethRevilla, Raquel GarcíaFogacci, FedericaReybrouck, MarkFormenti, DamianoRezania, ShahabaldinFotia, LidiaReznik, Jacqueline E.Fowler, LaurenRiccò, MatteoFrade, João Manuel GraçaRichmond, JanetFrancis, LynRifkin, SusanFrancisci, SilviaRiley, Carley L.Freeman, LauraRinninella, EmanueleFrei, ThomasRios-Figueroa, Homero VladimirFriedson, AndrewRistić-Medić, DanijelaFriesen, Craig A.Rivinius, RasmusFujioka-Kobayashi, MasakoRivolta, Massimo WalterFunk, RichardRizzo, GianlucaGablonski, Thorsten-ChristianRobinson, Monica L.Gabryś, TomaszRobres, AlbertoGagné, IsaacRodrigues-de-Souza, Daiana PriscilaGaire, RajRodríguez, Carlos FreireGaláž, ZoltánRodriguez-Arrastia, MiguelGaleazzi, Gian MariaRodríguez-Hurtado, DianaGallego-Sendarrubias, Gracia MaríaRodríguez-Rojo, Inmaculada ConcepciónGanzfried, SamRoehrich, JensGaragiola, UmbertoRogozea, LilianaGarcez Sardo, Pedro MiguelRojas-Khalil, YeseniaGarcía Navarro, Esperanza BegoñaRojo-Suárez, JavierGarcia, AmberRomero Martínez, ÁngelGarcía, FernandoRomero Rodríguez, José MaríaGarcía, YolandaRomero, Laura Ponce De LeónGarcia-Constantino, MatiasRomero-Galisteo, RitaGarcía-Pola, María JoséRomero-Saldaña, ManuelGarrett, Mario D.Ronnebaum, JulieGash, HughRoque, VitorGasparri, RobertoRosales-Mayor, EdmundoGazerani, ParisaRossi, AmerigoGe, WenzhenRowe, PeterGerbershagen, Mark UlrichRoy, DipankarGerke, OkeRubio-Arraez, S.Germain, IsabelleRubira-García, RainerGermanotta, MarcoRudnick, AbrahamGhassib, IyaRudnytskyi, IegorGholamipour-Shirazi, AzarmidokhtRudrappa, MohanGiannella, LucaRuiz-Palmero, JulioGibbons, JeffRupp, Jonathan D.Gibuła-Tarłowska, EwaRustico, LorenzoGiedraitis, VincentasRuzieh, MohammedGijón-Nogueron, GabrielRyabov, IgorGijsberts, Marie-José H.E.Ryder, SheilaGissler, MikaRykkje, LindaGiuffrè, MauroRyskulova, AselGlass, SteveRzońca, EwaGoldsweig, Andrew M.Saad, MarceloGomes, Joao RSaad, Nizar Y.Gómez-de-Terreros-Guardiola, MontserratSadak, Karim ThomasGómez-Salgado, JuanSaha, Amit KumarGómez-Urquiza, Jose L.Saini, ShikhaGoniewicz, MariuszSaitoh, TakejiGonzalez Arteaga, TeresaSaiz Galdós, JesúsGonzález Valero, GabrielSaksvik-Lehouillier, IngvildGonzález, CarinaSalerno, SergioGonzález, EvelioSalichs, EstherGonzalez-Rodriguez, AlexandreSalinet, JoaoGraça, Flávia AparecidaSambri, AndreaGrande, FrancescoSamorinha, CatarinaGray, Janet M.Sánchez Loyo, Luis MiguelGreenberg, JonathanSanchez-Gordon, SandraGreenberg, RosalieSanchez-Romero, Jose-LuisGreetham, DarrenSantabarbara, J.Gregório, JoãoSantander-Jiménez, SergioGrewal, Raji PaulSantiago Junior, Joel FerreiraGrimm, Wolf-DieterSantillan Garcia, AzucenaGude, FranciscoSantinha, GonçaloGuirguis, AmiraSantos, Josefa GonzálezGulis, GabrielSantulli, GaetanoGunasekaran, KulothunganSanz, AlejandroGuo, MingchunSapezinskiene, LaimaGupta, VikasSaravanakumar, KandasamyGuriec, NathalieSarfraz, MuddassarGurwitt, AlanSarnovsky, MartinGutiérrez-Manzanedo, José V.Sase, SunetraHain, RichardSaturnino Lupi, MarcoHakeem, Faisal F.Sauer, Pieter JjHamadi, Hanadi YSchlenke, PeterHamano, TadanoriSchopfel, JoachimHamilton, LuzaanSchultz, DavidHammond, WilliamSchumm, Walter R.Han, YuyanSchuurman, Michiel J.Hara, TakatoshiScicchitano, PietroHartung, JohnScraba, Paula J.Hashemi, ShervinScuri, StefaniaHassan, Amr Abdulfattah HausienSedikki, LyndaHattink, BartSeeman, Mary V.Hausien, AmrSegura-Ortí, EvaHealy, AoifeSehgal, RahulHébert, JohanneSejdic, ErvinHeinz, AndreasSemova, IvanaHeller, DeboraSemwal, MonikaHelsloot, IraSengupta, ArjunHempel, GuntherŞenocak, ÇağrıHerbert, AnthonySeo, Dong-OhHermann, CarollSerrano Rosa, Miguel AngelHernández-Pérez, IvánSerrano-Gomez, DiegoHerrera-López, Enrique J.Serra-Paya, NoemiHerrera-Peco, IvánServidio, Rocco CarmineHerrero De La Parte, BorjaSettembre Blundo, DavideHerrero, María JesúsShaffner, Donald H.Hess, MoritzShakya, AkhileshHesse, MortenShamionov, Rail M.Heston, Thomas FShamputa, Isdore CholaHewson, DavidShan, Xiaojun (Gene)Hilton, Thomas F.Sharma, GauravHingorani, RittuSharma, RavindraHo, RogerShaver, Amy L.Hoerr, RobertShelley, MackHofer, Matthias D.Shen, JunHoffer, Edward P.Sheng, JianxiongHolwerda, Seth W.Sherman, PaulHoran, DavidSherratt, R. SimonHort, KrishnaShi, YushengHoshi, Rosangela AkemiShin, Hyoung ChulHosie, AnnmarieShin, Won-SopHossain, Syeda ZakiaShindova, MariaHostiuc, SorinShon, Ho SunHou, RuixueSidorov, EvgenyHou, XiaorongSilva, JoséHuang, QijunSilva, SamuelHuang, Yen-MingSilvestri, GiovanninoHuda, Fauzia AkhterSimón-López, Leticia CarmenHugelius, KarinSingh, Pankaj KHung, Chia-LiangSingh, ParamHunter, RachaelSioofy-Khojine, AmirbabakHuq, Mona SayedalSiopis, GeorgeHuseth-Zosel, Andrea L.Siristatidis, CharalamposHutton, VickiSistla, SrinivasHyun, EunjungSkullerud, JonivarIacoviello, MassimoSkurikhin, Evgenii GermanovichIbáñez-Costa, AlejandroSnell, ChristopherIbáñez-Vera, Alfonso JavierSocea, BogdanIdler, Ellen L.Sokang, Yasinta AstinIhle, AndreasSolomon, NancyIlias, IoannisSoma, MasayukiIlyas, MohammedSon, YeonjungImai, TakaoSonne, James W.Imtiaz, MohammadSousa, AntónioInoue, KatsufumiSouza, MargaretInuso, GiuseppinaSpanakis, Emmanouil G.Inzani, FredianoSpeight, NigelIrusta, UnaiSpencer, LissaIsmael, SaifudeenSperlì, GiancarloItatani, TomoyaSrouji, SamerIto, KanadeStachowska, EwaIvanitskaya, LanaStaines, DonIwai, ToshinoriStalsberg, HelgeIzawa, Kazuhiro P.Stambolieva, MarijaIzeta, AnderStavropoulou, AretiIzonin, IvanStefanini, AlessandroIzydorczyk, BernardettaStokmans, MiaJaggavarapu, SiddharthStorsberg, JoachimJakovljevic, MihajloStrametz, ReinhardJakovljevic, Mihajlo B.Stretesky, PaulJames, LoisStricker, RaphaelJang, Sou HyunStringham, JamesJankowski, MateuszStripay, JenniferJatužis, DaliusStrohfus, Pamela K.Javier Rodríguez Hernández, HumbertoStronge, PaulJaworowska, AgnieszkaSuchý, JiříJeong, Keun-YeongSudraba, VelgaJiang, XuejuanSuehiro, TadanobuJillella, DineshSukys, SauliusJimenez, DianaSun, Kai SingJimenez, RicardoSuominen, Sakari B.Jimenez-Rejano, José-JesúsSurdacki, AndrzejJo, SunmoonSuryavanshi, SantoshJoda, TimSutton, JillJohansson, EdvardSvenson, LarryJokela, MeritaSvensson, AnnJones, Corinne A.Świetlicka, IzabelaJordao, LuisaSyed, IffathJozwiak, MathieuSygit, KatarzynaJuárez-Vela, RaúlSzczepanek, JoannaJudzińska, AnetaSzczesniak, DorotaJung, Bock-YoungSzcześniak, MałgorzataJung, Jin-HwaSzlagatys-Sidorkiewicz, AgnieszkaKaden, MarikaSzromek, AdamKaesmann, LukasTaber, Christopher B.Kalus, AlicjaTaborri, JuriKamenidou, Irene (Eirini)Tada, AkioKampantais, SpyridonTajiri, NaokiKang, Gu EonTakahashi, KazutakaKantake, MasatoTakeda, HiroyukiKantor, JiříTalavera, MartaKao, Li-TingTanaka, Tim HideakiKapan, AliTange, H.J.Kapočiūtė-Dzikienė, JurgitaTannous, KathyKarim, JahangirTanoubi, IssamKarniej, PiotrTarantino, GiovanniKasten, ErichTarazona, FranciscoKatina, PolinpapilinhoTaylor, LynneKaurani, LalitTecco, SimonaKaye, ElizabethTender, Gabriel ClaudiuKeidar, OsnatTeramoto, ShinjiKellar-Guenther, YvonneTestoni, InesKeller, BetsyThai, YvonneKennedy, GerardThangathurai, DuraiyahKessler, DannyThirunavukkarasu, ShyamalaKevorkian, MelineThornes, John EdwardKhacharem, AïmenTiberius, VictorKhan, HamidullahTinney, GlendaKhan, WasiqTiseo, CarloKharat, SuhasTolhurst, EdwardKhashayar, PatriciaTomaszewska, EwaKhemani, Robinder G.Tompkins, ChristopherKhezr, SeyednimaTomsone, SigneKholová, IvanaTonacci, AlessandroKhor, YetTong, Yat-ChingKhor, Yet HongTorres, Olga LopezKhuder, Sadik A.Torrico, DamirKico, IrisToru, IchisekiKilner, TimTramontano, MarcoKim, Amy Chan HyungTrankle, StevenKim, Beom KyungTranstrum, Mark K.Kim, ForestTremolada, MartaKim, George R.Tripp, LianneKim, Hae-YoungTseng, Hsiao-TingKim, Hye LyunTsourtos, GeorgeKim, HyeongsuTudor, Mihaela-AlexandraKim, JieunTuretta, MatteoKim, Jin-BomTurk, MelanieKim, JungWookTurner, Arlener D.Kim, Nam-GyoonTyagi, MuditKim, NamkwenUchida, OsamuKim, Ryong NamUnderdahl, LouiseKim, SangjinUnger, ElizabethKim, Su HyunUseche, Sergio A.Kim, SunheeVaccaro, Joan A.Kim, Young-JaeValente, Nicola AlbertoKimura, MasahiroValenzuela, RodrigoKing, ChristianValicherla, Guru RaghavendraKirchen, TwyllaValkonen, Tarmo JohannesKirkegaard, Emil O. W.Valvani, RachnaKiselev, JörnVan Der Borg, JanKislitsina, OlgaVan Der Heijden, JeroenKiss, MariettaVan Dijk, Jitse P.Kitsios, FotisVan Overmeire, RoelKlapdor, RüdigerVan Schaik, Katherine D.Klemens, BrygidaVance, David E.Klimek, MarkusVaradinov, Maria JoséKlukowska-Rötzler, JolantaVarela, GonzaloKnechtle, BeatVarghese, MathewsKnoll, Eva-MariaVashist, Sandeep KumarKo, JesukVavrek, RomanKocot, EwaVázquez Lara, Juana MaríaKodama, KotaVecellio, EliaKodate, NaonoriVellone, Valerio GaetanoKohara, YukihiroVenetsanou, FotiniKokubun, KeisukeVergo, Maxwell TKomnenov, DraganaVerso, Maria GabriellaKones, RichardVezzani, BiancaKonstantinov, VsevolodVicente, SandovalKontsevaya, IrinaVick, DanKoo, MalcolmVimarlund, VivianKopsaftis, ZoeVincent, GraceKormuth, KarenVink, MarkKörner, AnitaVirgolino, AnaKos, MarkoVišnjić, AleksandarKotsanos, NikolaosVitolo, Marcia ReginaKou, GangVlachou, EugeniaKoubassova, NataliaVon Visger, Tania T.Koukouli, SofiaVuorio, AlpoKowalczyk, TomaszWada, JunKowalewski, MaciejWalheer, BarnabéKoyama, ShihokoWalker, MarvinKrueger, EddyWalton-Moss, Benita JeanneKrutsinger, Dustin CWan, Thomas T. H.Kudryashova, Tatiana V.Wang, Chin-TienKumar, AmitWang, PengchengKumara, Ajantha SisiraWang, QunKumble, Peter A.Wang, RuijieKume, KensukeWang, XishaKump, DavidWard, MarkKuo, YiliangWashburn, David J.Kupcewicz, EwaWatanabe, YoichiKuppusamy, ManiselvanWeaver, JolantaKuryłowicz, AlinaWebster, Craig StephenKwon, Yu-jinWhite, JanineLafave, Lynne M.Z.Whitehead, Patrick M.Lai, YuanWieczorek, AnetaLam, Simon ChingWilliamson, Ryan D.Lamb, ChristopherWills, MelanieLang-Roth, RuthWilson, Christopher G.Lapré, FreekWilson, LeighLaranjeira, CarlosWindsor, FredricLashley, MaryWinston, Bruce E.Lasker, JordanWolska, MałgorzataLaverack, Glenn R.Wood, Kelly S.Lawrence, MaggieWorkman, Craig D.Lazarus, SulemanWu, Chiao-EnLeal Costa, CesarWu, HannahLedda, CaterinaWu, JiankangLee, Alvin J. X.Wu, Tzu-HuaLee, Chang WonWu, WenchenLee, ChanghoonWysokinski, MariuszLee, Hyoung YongXiao, ZhiMinLee, Hyun JinXu, HuiLee, Jae HoXu, KailiLee, JemyungYamamoto, MiwaLee, JongHyukYamatsu, KojiLee, Kyoung JinYamazaki, TatsuyaLee, LizaYang, Nan-PingLee, PeterYang, XiaoLee, WonYang, ZanLee, WooinYao, YuChunLee, Wui-ChiangYeung, Timothy Yu-CheongLee, Young-SilYokomichi, HiroshiLefley, FrankYoo, YongjaeLeicht, Kevin T.Yu, RondyLeimanis, MaraYue, TaoLemoyne, SabineZagórska, AgnieszkaLenzi, JacopoZakrocka, IzabelaLeo, Carlo GiacomoZalewska, MagdalenaLeón-Quismondo, JairoZaltauskaite, JurateLesiuk, TeresaZanini, MilkoLeslie, MonicaZechariah, SunithaLeszczyński, PiotrZegrean, MihaelaLeung, SuzannieZhang, DuoLew, EileenZhang, HuifengLi, HanchuanZhang, ZhenLi, JianxinZheng, XuyuanLi, LiZhu, Bo-WeiLi, ManyuZhu, YongLi, ShuoZiada, HassanLi, XiuyangZimmermann, AgnieszkaLidbury, BrettZolotukhin, Igor A.Liegner, KennethZumeta, William M.Li-Jessen, NicoleZweiker, DavidLiller, Karen

